# Role of radiomics in staging liver fibrosis: a meta-analysis

**DOI:** 10.1186/s12880-024-01272-x

**Published:** 2024-04-12

**Authors:** Xiao-min Wang, Xiao-jing Zhang

**Affiliations:** 1 School of Medical Imaging, Tianjin Medical University, No.1, Guangdong Road, Hexi District, Tianjin, 300203 China; 2 Department of Radiology, The First Medical Center, Chinese PLA General Hospital, Beijing, 100853 China

**Keywords:** Radiomics, Liver, Fibrosis, Cirrhosis, MRI, CT, Ultrasound

## Abstract

**Background:**

Fibrosis has important pathoetiological and prognostic roles in chronic liver disease. This study evaluates the role of radiomics in staging liver fibrosis.

**Method:**

After literature search in electronic databases (Embase, Ovid, Science Direct, Springer, and Web of Science), studies were selected by following precise eligibility criteria. The quality of included studies was assessed, and meta-analyses were performed to achieve pooled estimates of area under receiver-operator curve (AUROC), accuracy, sensitivity, and specificity of radiomics in staging liver fibrosis compared to histopathology.

**Results:**

Fifteen studies (3718 patients; age 47 years [95% confidence interval (CI): 42, 53]; 69% [95% CI: 65, 73] males) were included. AUROC values of radiomics for detecting significant fibrosis (F2-4), advanced fibrosis (F3-4), and cirrhosis (F4) were 0.91 [95%CI: 0.89, 0.94], 0.92 [95%CI: 0.90, 0.95], and 0.94 [95%CI: 0.93, 0.96] in training cohorts and 0.89 [95%CI: 0.83, 0.91], 0.89 [95%CI: 0.83, 0.94], and 0.93 [95%CI: 0.91, 0.95] in validation cohorts, respectively. For diagnosing significant fibrosis, advanced fibrosis, and cirrhosis the sensitivity of radiomics was 84.0% [95%CI: 76.1, 91.9], 86.9% [95%CI: 76.8, 97.0], and 92.7% [95%CI: 89.7, 95.7] in training cohorts, and 75.6% [95%CI: 67.7, 83.5], 80.0% [95%CI: 70.7, 89.3], and 92.0% [95%CI: 87.8, 96.1] in validation cohorts, respectively. Respective specificity was 88.6% [95% CI: 83.0, 94.2], 88.4% [95% CI: 81.9, 94.8], and 91.1% [95% CI: 86.8, 95.5] in training cohorts, and 86.8% [95% CI: 83.3, 90.3], 94.0% [95% CI: 89.5, 98.4], and 88.3% [95% CI: 84.4, 92.2] in validation cohorts. Limitations included use of several methods for feature selection and classification, less availability of studies evaluating a particular radiological modality, lack of a direct comparison between radiology and radiomics, and lack of external validation.

**Conclusion:**

Although radiomics offers good diagnostic accuracy in detecting liver fibrosis, its role in clinical practice is not as clear at present due to comparability and validation constraints.

**Supplementary Information:**

The online version contains supplementary material available at 10.1186/s12880-024-01272-x.

## Introduction

Chronic liver disease is an important health concern due to high prevalence of metabolic dysfunction associated fatty liver disease (MAFLD), hepatitis B/C, and alcoholic liver disease. Whereas mortality due to liver disease has declined in some countries like the USA and China, some countries such as India and Mongolia still have higher mortality rates. Increasing trends in the prevalence are also noted in the United Kingdom and Russia [1]. In the USA, 1.8% of the population has a liver disease diagnosis [2]. In China, although the mortality rates have decreased, the prevalence of liver disease is increasing [3]. Globally, the incidence of non-alcoholic steatohepatitis-caused cirrhosis is increasing by 1.35% each year [4].

Liver fibrosis is a modifiable factor that is associated with worse health outcomes, transplants, and mortality [5]. Liver fibrosis may develop due to chronic viral infection, long-term alcohol use, or steatohepatitis. It is estimated that liver fibrosis affects 7.7% of individuals in the general population of the United States of America and obese individuals are at much higher risk [6]. The latest stage of fibrosis, cirrhosis, is the eleventh leading cause of mortality [1]. An increasing trend in mortality due to cirrhosis has been observed globally from 1990 to 2017 [7]. Several methods are available to measure liver fibrosis. The liver biopsy is considered diagnostic “gold standard” for staging liver fibrosis. The biopsy is a highly valuable tool but can be associated with complications, sampling errors, and between-observer variations in judgments. Fibrosis can be patchy so that not all parts of the liver contain fibrosis evenly, therefore, a biopsy may fail to capture some samples [8]. Among other methods, serum biomarkers may also help in detecting fibrosis. Radiological methods including magnetic resonance imaging (MRI) magnetic resonance elastography (MRE), computed tomography (CT), ultrasonography, and elastography also provide non-invasive means of measuring fibrosis [9].

Radiomics is a post-radiology process of high-throughput extraction of features from radiological images for conversion into mineable data involving complex processes of artificial intelligence such as machine learning, deep learning, and convolutional neural networks to maximize predictability. It is developed on the premise that pathophysiological tissues and organs contain much information that can be quantified and differentiated from normal tissues and organs. Extraction of a large number of features to form a database and then mining the data for analyses aids decision support leading to improved diagnostic accuracy and prognostic capability [10–12]. Radiomics takes texture as a spatial arrangement of predefined voxels through which complicated features of the image can be read and mathematical calculations of these arrangement characteristics differentiate normal from abnormal. The heterogeneity in the selected features reflects the heterogeneity of histopathological changes [13]. Radiomics features can be morphological, histogram, textural, and high-order features. Morphological features include the shape, size, and volume of the region of interest. Histogram is the plotting of pixel values against pixel frequency and can be used to describe many features such as magnitude, dispersion, asymmetry, peakedness, flatness, randomness, uniformity, etc. Textural features provide spatial relationships between neighboring pixels. High-order features are those acquired after applying filters to images [12, 14].

Several reviews have described the role of radiomics in the diagnosis and staging of various types of cancers [15, 16]. Among other clinical applications, radiomics has been found to be a valuable aid in cardiomyopathy [17, 18], musculoskeletal diseases [19, 20], neurological and psychiatric disorders [21–24], and liver diseases [25]. Several studies have reported the diagnostic accuracy indices of radiomics in diagnosing and staging liver fibrosis. However, there is no synthesis of these outcomes which are sometimes variable and even inconsistent. The present study aimed to evaluate the role of radiomics in diagnosing and staging liver fibrosis by conducting a systematic review of relevant studies and performing meta-analyses of statistical indices.

## Method

The present study was conducted by following PRISMA guidelines.

### Inclusion and exclusion criteria

A study was included in the meta-analysis if a) it prospectively or retrospectively recruited patients with chronic liver disease who had histologically confirmed fibrosis in the liver; b) performed radiomic analyses based on any radiological modality to diagnose and/or differentiate fibrosis; and c) reported diagnostic accuracy indices of radiomics in diagnosing and differentiating liver fibrosis stages by in comparison with histopathology. Studies were excluded based on the following criteria: a study a) reported diagnostic performance of radiomics for liver fibrosis without adequate statistical data; b) reported the outcomes of pediatric patients; and c) reported the diagnostic accuracy of a combined clinical-radiological radiomics model.

### Literature search

Electronic scientific databases (Embase, Ovid, Science Direct, Springer, and Web of Science) were searched for the identification of relevant studies using area-specific keywords. The primary search strategy was “Radiomics AND liver fibrosis OR cirrhosis AND diagnostic accuracy”. Secondary keywords were used in several other combinations with this primary string. The detailed literature search strategy is given in Appendix S1. After the identification of studies, reference lists of related articles were also screened for additional studies. The literature search encompassed peer-reviewed research articles published in English from the date of database inception till May 2023.

### Data analysis

Data on the design and conduct of studies, patient demographics, clinical characteristics, fibrosis stage, radiomics design and analyses, and diagnostic accuracy outcome data were extracted from the research articles of respective studies and organized in data sheets. The quality of the included studies was assessed with the Quality Assessment of Diagnostic Accuracy Studies (QUADAS-2) scale. This scale assesses the quality of studies under the domains of risk of bias and applicability concerns by evaluating patient selection, index test, reference standard, and flow and timing. Diagnostic accuracy endpoints (Accuracy, area under receiver operator curve (AUROC), sensitivity, and specificity of various radiomics models compared to histologically proven fibrosis were extracted from the research articles of respective studies and pooled under random effects model using the point estimates and their 95% confidence intervals of these indices. Subgroup analyses were performed with respect to fibrosis stage (significant fibrosis; stages F2-4, advanced fibrosis; stages F3-4, and cirrhosis; stage F4) and with respect to the study cohort (training, test/validation). Statistical analyses were performed with Stata software (Stata Corporation, College Station, Texas, USA).

## Results

**Fig. 1:**
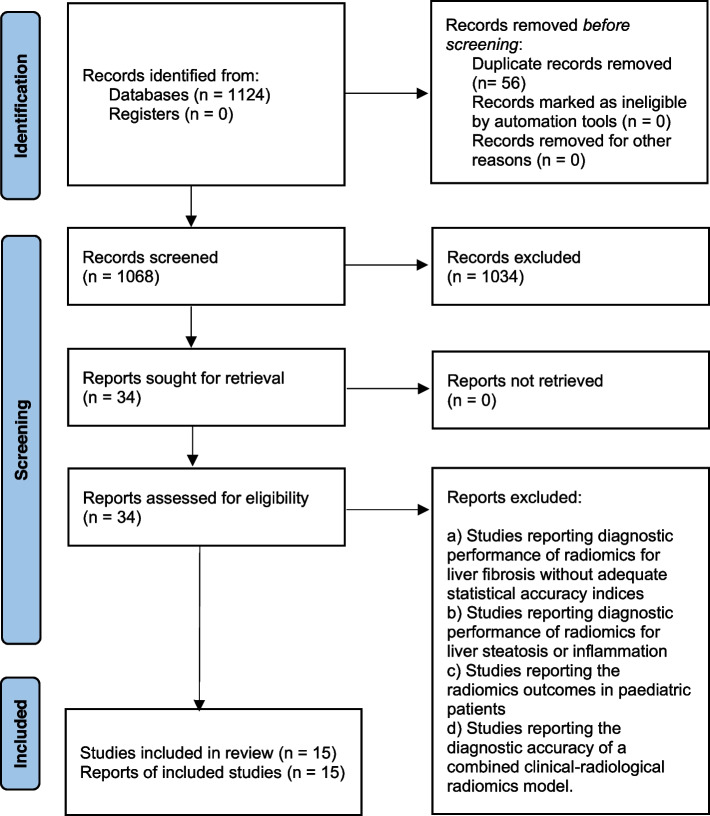
A flowchart of study screening and selection process

Fifteen studies [13, 26–39] were included that were published between 2018 and 2023 (Fig. 1). In these studies, radiomic analyses were performed involving 3718 patients with chronic liver diseases including hepatitis and MAFLD. The age of these patients was 47.3 years [95% confidence interval (CI): 42.0, 52.5]. The proportion of males was 69% [95% CI: 65, 73]. Histologically (biopsy/surgical) confirmed fibrosis stage was F0 in 13% [95% CI: 7,20], F1 in 17% [95% CI: 13, 23], F2 in 21% [95% CI: 17,26], F3 in 16% [95% CI: 13,19], and F4 in 27% [95% CI: 23, 33] of the patients. Radiological modalities used in these studies were: MRI (5), CT (3), ultrasonography (2), positron emission tomography (1), shear-wave elastography (2), and MRE (2).

Important characteristics of the included studies are given in Table S1. The quality of the included studies was moderate in general according to the QUADAS-2 scale (Table S2). The risk of bias was limited to the retrospective design of studies which could have introduced patient selection bias. Moreover, the interval between the index test and reference test was up to six months which might have impacted adequate flow and timing. Included studies also varied with regards to validation ranging from no validation [26], Five/Ten-fold cross-validation [27, 30, 32, 36, 39], and leave-one-out cross-validation [13] to internal validation [28, 30, 31, 33, 34, 37, 38], and internal and external validation [29]. There was no significant publication bias according to Egger’s test (Bias coefficient 0.114 [-11.80, 12.03]; *p*=0.984) or Begg’s test (Adjusted Kendall’s score -29±18; *p*=0.125) (Figure S1a and b).

**Fig. 2:**
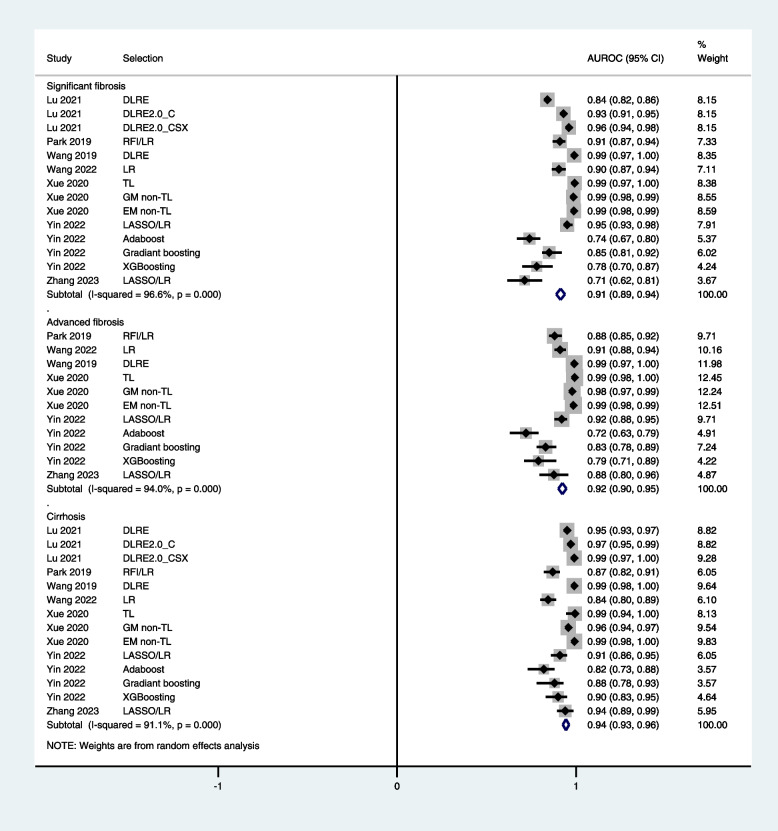
Forest graphs showing the outcomes of meta-analysis of AUROC values of radiomics in diagnosing fibrosis stages in training cohorts

**Fig. 3:**
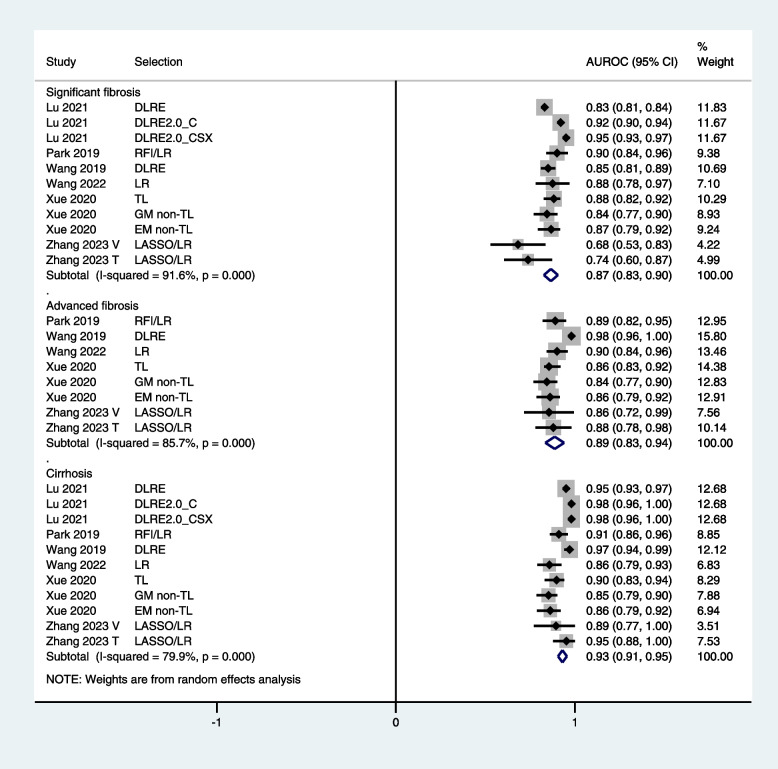
Forest graphs showing the outcomes of meta-analysis of AUROC values of radiomics in diagnosing fibrosis stages in test/validation cohorts

The pooled AUROC value of radiomics for the diagnosis of any liver fibrosis was 0.878 [95% CI: 0.850, 0.906]. The AUROC values for the detection of significant fibrosis, advanced fibrosis, and cirrhosis were 0.914 [95% CI: 0.889, 0.938], 0.924 [95% CI: 0.901, 0.946], and 0.944 [95% CI: 0.929, 0.963] respectively in training cohorts, and 0.886 [95% CI: 0.826, 0.905], 0.887 [95% CI: 0.834, 0.939], and 0.930 [95% CI: 0.905, 0.954] respectively in test/validation cohorts (Figs. 2 and 3).

The pooled accuracy of radiomics in diagnosing any liver fibrosis was 83.5% [95% CI: 81.7, 85.4]. The accuracy values of radiomics in the detection of significant fibrosis, advanced fibrosis, and cirrhosis were 80.6% [95% CI: 76.2, 85.1], 83.5% [95% CI: 81.3, 85.8], and 81.6% [95% CI: 78.7, 84.5] respectively in training cohorts, and 77.0% [95% CI: 68.6, 85.5], 84.3% [95% CI: 79.8, 88.8], and 81.3% [95% CI: 77.0, 85.7] respectively in test/validation cohorts (Figures S2a and b).

**Fig. 4:**
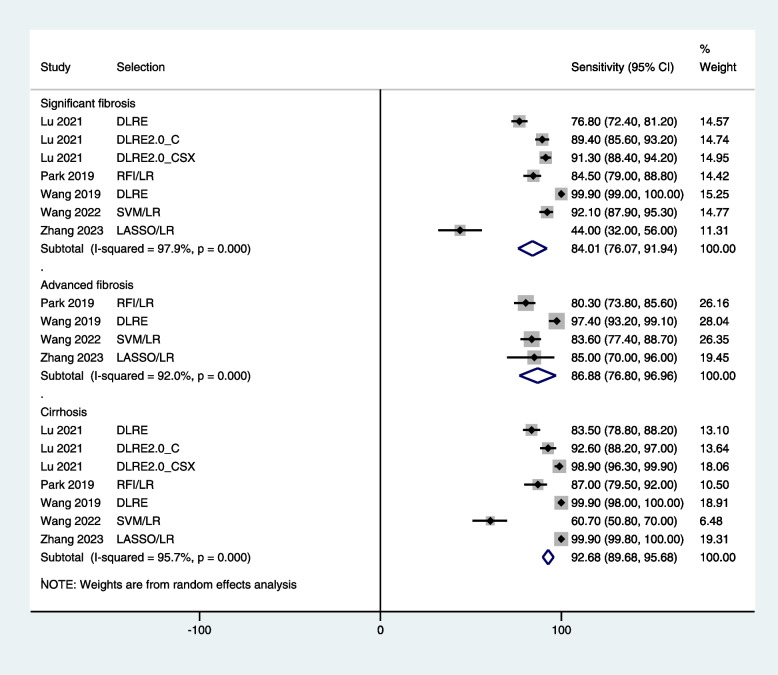
A forest graph showing the outcomes of meta-analysis of the sensitivity of radiomics in diagnosing fibrosis stages in training cohort

**Fig. 5:**
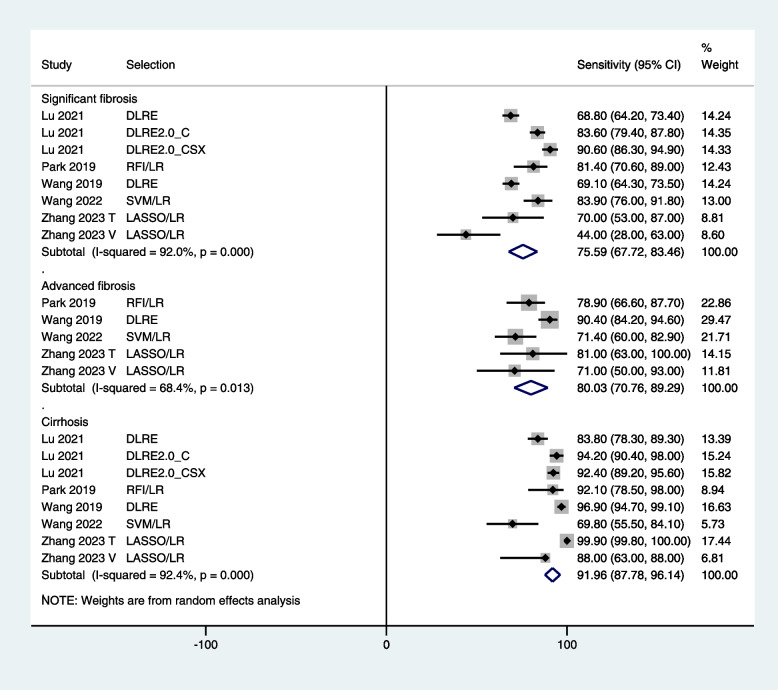
A forest graph showing the outcomes of meta-analysis of the sensitivity of radiomics in diagnosing fibrosis stages in test/validation cohorts

In training cohorts, the sensitivity of radiomics in diagnosing significant fibrosis, advanced fibrosis, and cirrhosis was 84.0% [95% CI: 76.1, 91.9], 86.9% [95% CI: 76.8, 97.0], and 92.7% [95% CI: 89.7, 95.7] respectively (Fig. 4). In test/validation cohorts, the sensitivity of radiomics in diagnosing significant fibrosis, advanced fibrosis, and cirrhosis was 75.6% [95% CI: 67.7, 83.5], 80.0% [95% CI: 70.8, 89.3], and 92.0% [95% CI: 87.8, 96.1] respectively (Fig. 5).

In training cohorts, the specificity of radiomics in diagnosing significant fibrosis, advanced fibrosis, and cirrhosis was 88.6% [95% CI: 83.0, 94.2], 88.4% [95% CI: 81.9, 94.8], and 91.1% [95% CI: 86.8, 95.5], whereas in test/validation cohorts, the specificity was 86.8% [95% CI: 83.3, 90.3], 94.0% [95% CI: 89.5, 98.4], and 88.3% [95% CI: 84.4, 92.2] respectively (Figure S3a and b).

Among the included studies, Hu et al. [27] who found thin-sliced CT images to yield better performance of radiomics than thick-sliced CT images reported AUROC values of 0.90 [95% CI: 0.84, 0.96] for F1 vs F2-4, 0.85 [95% CI: 0.78, 0.92] for F1-2 vs F3-4, and 0.94 [95% CI: 0.89, 0.97] for F1-3 vs F4 stages. Lan et al. [13] who studied MRE radiomics found AUROC values of 0.89 [95% CI: 0.84, 0.94] for F0 vs F1-4, 0.93 [95% CI: 0.89, 0.98] for F0-1 vs F2-4, 0.92 [95% CI: 0.88, 0.97] for F0-2 vs F3-4, and 0.95 [95% CI: 0.90, 0.997] for F0-3 vs F4 stages in their echo-planar images. They observed AUROC values of 0.89 [95% CI: 0.83, 0.94], 0.87 [95% CI: 0.81, 0.93], 0.89 [95% CI: 0.84, 0.95], and 0.94 [95% CI: 0.89, 0.997] for F0 vs F1-4, F0-1 vs F2-4, F0-2 vs F3-4 and F0-3 vs F4 stages respectively in their gradient recalled echo images.

## Discussion

This meta-analysis found that radiomics exhibits high accuracy in diagnosing and staging liver fibrosis. The AUROC values for the detection of significant fibrosis, advanced fibrosis, and cirrhosis were approximately 0.91, 0.92, and 0.94 in training cohorts and 0.89, 0.89, and 0.93 in validation cohorts, respectively. However, despite the good efficiency of radiomics in diagnosis and staging liver fibrosis observed herein, several factors make these findings inconclusive and dependent on future studies to refine this evidence. For example, several types of radiological modalities were used for radiomics in individual studies and a direct comparison of diagnostic performance between radiomics and radiology was mostly lacking. Moreover, various models were utilized for radiomics, and these studies lacked external validation.

Some studies that could not be included in this meta-analysis have also reported variable diagnostic performance of radiomics in staging liver fibrosis. Cui et al. [40] utilized multiphase CT-based radiomics to stage liver fibrosis and found the sensitivity of diagnosing significant fibrosis, advanced fibrosis, and cirrhosis to be 30-41%, 59-71%, and 84-87%, and the specificities being 84-90%, 71-79%, and 50-58% respectively. Duan et al. [41] observed better performance of ultrasound-based radiomics in diagnosing cirrhosis (AUROC 0.86) than advanced fibrosis (AUROC 0.77). Zhou et al. [42], also reported higher efficieny of ultrasound-based radiomics in diagnosing cirrhosis (AUROC 0.83-0.86) than significant fibrosis (AUROC 0.69-0.71) or advanced fibrosis (AUROC 0.67-0.72).

Many studies have shown that the diagnostic accuracy of radiomics is better than serological biomarkers [30, 42–44]. Sim et al. [32] found better diagnostic performance of MRE radiomics (AUROC 0.97 [95% CI: 0.93, 1]) than clinical features (AUROC 0.91 [95% CI: 0.81, 0.97]) in diagnosing significant fibrosis. Wang et al. [34] also reported that the AUROC values for significant fibrosis, advanced fibrosis, and cirrhosis were higher with CT-based radiomics (AUROC 0.88, 0.90, and 0.86) than with either aspartate transaminase-to-platelet ratio (AUROC 0.69, 0.67, and 0.65) or Fibrosis-4 index (AUROC 0.71, 0.71, and 0.7) respectively. Xue et al. [35] also found better diagnostic performance of multimodal ultrasound radiomics (AUROC 0.9-0.93) than either aspartate transaminase-to-platelet ratio (AUROC 0.72-0.78) or Fibrosis-4 index (AUROC 0.69-0.75) in staging liver fibrosis. Zhao et al. [38] found better diagnostic efficiency of MRI radiomics (accuracy 0.8) than clinical markers (accuracy 0.68) in differentiating non-significant fibrosis from clinically significant fibrosis in the test cohort. Some authors have suggested that a model combining radiomics and clinical biomarkers may further improve the diagnostic accuracy of fibrosis [38, 45].

Despite good diagnostic accuracy values observed for radiomics in liver fibrosis assessment in the present study, inconsistencies were observed in the outcomes of individual studies. Whereas Wang et al. [34] and Lan et al. [13] found radiomics better than radiological elastography in diagnosing advanced fibrosis and cirrhosis, Sim et al. [32] did not find a statistically significant difference. Lu et al. [29] and Sim et al. [32] found that radiomics distinguished well between significant fibrosis (F2-4) and non-significant fibrosis (F0-1). However, Zhang et al. [37] reported that radiomics was unable to distinguish between non-significant fibrosis and significant fibrosis. Zhao et al. [38] reported that a combined use of radiomics and clinical biomarkers performed better than radiomics alone, whereas Sim et al. [32] did not find a significant difference in performance between radiomics and combined use of radiomics, radiology, and clinical biomarkers.

We have observed that some diagnostic accuracy indices were slightly higher in training cohorts than in validation cohorts. Wang et al. [33] who found that the accuracy of deep learning radiomics of elastography for the diagnosis of significant fibrosis was lower in the validation cohort in comparison with the training cohort suggested that this could be because of the lower heterogeneity in F0 and F1 groups and can be overcome possibly by adapting multiple strategies for fibrosis classification. Lu et al. [29] found that AUROC values increased for their deep learning radiomics of elastography when datasets had a higher prevalence of patients with F0 and F1 stages. They suggested that the inclusion of higher proportions of patients with F0 and F1 stages can yield better accuracy because about 80% of patients with chronic hepatitis B have F0 or F1 stage in the general population. In the present study, the pooled percentages of patients with F0 and F1 stages were 13% and 17% respectively.

Fibrosis develops by the excessive deposition of extracellular matrix in the liver as a response to wound healing after which satellite cells activate, higher levels of alpha smooth muscle actins are produced, and collagen I/II are synthesized. Such processes increase the stiffness of the liver progressively and may lead to cirrhosis. Fibrosis is usually associated with the accumulation of collagen fibers, not well-defined portal vein walls, and irregular hepatic vein margins. Gray-scale ultrasound images capture such information to reflect the scattering of fine structures. Moreover, coarse echotexture and a mild increase in echogenicity of hepatic parenchyma are usually observed in cirrhosis [35].

So far, studies addressing radiomics lack robust validation in larger and clinically diverse settings which present reproducibility challenges. In the present review, we found that all except one study involved internal validation, and therefore, the synthesis of these outcomes remains inconclusive and dependent on future studies with larger sample sizes and better designs with special focus on external validation. Radiomics models without external validation are at increased risk of being specialized in specific radiographs that hamper generalizability due to overfitting [46].

Overfitting and multi-collinearity may affect radiomics models. During training, high-dimensional features may overfit and thus may yield optimistic outcomes. Moreover, traditional statistical models may not work adequately to deal with multicollinearity among textural features. To avoid this, it is suggested that the removal of unreliable or irrelevant features and the reduction of dimensions of predictors may yield better outcomes [30, 31]. The sensitivity of AdaBoost to noisy data or outliers makes it more suitable for cases facing overfitting problems. A frequently used classifier, the Support Vector Machine, uses preselected nonlinear mapping to map input parameters in a high-dimensional feature space to optimize feature classification. Random forest unifies several weak predictor classifiers to make an accurate and stable predictor [28].

The AUROC is a performance metric to quantify the power of a model in discriminating cases from non-cases. An AUROC value can lie between 0 and 1. It combines the sensitivity and specificity of a marker/modality for the diagnosis of a precisely defined stage of fibrosis. Sensitivity is usually evaluated in patients with advanced fibrosis and specificity in non-advanced fibrosis [47]. However, the AUROC values can be biased if the fibrosis distribution in the study population differs from that of the whole population to which it is being applied [48]. Although biopsy is considered a gold standard for the diagnosis of fibrosis in the liver, it has a high rate of false positives and false negatives in comparison with the whole liver due to sampling error. An AUROC value of 0.82 for distinguishing between F2 and F1 when the entire liver was used as the reference index will inform approximately 20% error rate of the biopsy (false positive and false negative rates) compared with the entire liver. Thus, discordance in the staging of liver fibrosis between a modality such as radiomics and biopsy can be due to an error of the modality as well as due to an error of the biopsy [47].

Currently, the evidence regarding the role of radiomics in diagnosis of liver fibrosis and staging is constrained with several caveats. Quality of medical images acquired through different modalities may vary depending on several factors such as scanners, protocols, and personnel that can affect the reproducibility of radiomics output. A lack of standardization of image acquisition, preprocessing steps, extraction of features, and analyses also makes it difficult to compare radiomics outcomes of various studies performed under different settings. The etiology of fibrosis, progression, and the presence of comorbidities may also affect the accuracy of radiomics outcomes. Moreover, biological interpretation of radiomic features is lacking due to which it is difficult to associate radiomic features with histopathological characteristics.

Several limitations of the present study need to be considered while interpreting the outcomes of this review. An important limitation of the present study was the presence of high statistical heterogeneity in the meta-analyses. Although sources of heterogeneity could not be traced statistically, it is reasonable to assume that clinical and methodological heterogeneity might have played an influencing role. Authors utilized different methods for feature selection and classification, worked with a variety of software, and analyzed a highly variable number of features. Radiomics analyses were based on several radiological modalities and fewer studies were available to evaluate a particular modality in a pooled design. Most studies were retrospective in design due to which several types of biases could have been introduced. Inclusion and exclusion criteria differed substantially across the included studies that recruited several conditions of chronic liver disease including hepatitis B/C, autoimmune hepatitis, liver failure, early-stage cirrhosis, nonalcoholic fatty liver disease, and primary sclerosing cholangitis. Some studies could not be included because of the lack of variance data for diagnostic accuracy indices.

## Conclusion

In this meta-analysis of 15 studies, the use of radiomics in staging liver fibrosis has been found to be associated with good diagnostic accuracy. However, the present-day outcome data are inconclusive regarding the use of radiomics in clinical practice owing to heterogeneity in methodology and outcomes of reviewed studies in which the radiomic evaluations were based on several radiological modalities subjected to a variety of analytical models yielding varying outcomes and lacking external validation. Non-invasiveness and the involvement of machine learning make radiomics an attractive option for decision support. The outcomes reported so far are promising and need to be validated in multicenter studies having larger datasets and better comparability and validation aspects in designs.

### Supplementary Information

**Supplementary Material 1.**

## Data Availability

The datasets used or analyzed during the current study are available from the corresponding author on reasonable request.
