# Supplementary material for: Role of radiomics in staging liver fibrosis: a meta-analysis
Source: BMC Med Imaging. 2024 Apr 12;24:87. doi: 10.1186/s12880-024-01272-x (PMC11010385; doi:10.1186/s12880-024-01272-x)
Supplement: Supplementary file 1 — Supplementary Material 1. [file 12880_2024_1272_MOESM1_ESM.docx]

| **Table S1: Important characteristics of the included studies** | | | | | | | | | | | | | | | | |
| --- | --- | --- | --- | --- | --- | --- | --- | --- | --- | --- | --- | --- | --- | --- | --- | --- |
| **Study** | **n** | **Design** | **Patients** | **Age (Years)** | **Males (%)** | **BMI (Kg/m^2^)** | **Fibrosis stage** | | | | | **Radiomics characteristics** | | | | |
|  |  |  |  |  |  |  | **F0** | **F1** | **F2** | **F3** | **F4** | **Radiology** | **Software** | **Extracted features** | **Feature selection / classification approach** | **Important features** |
| Chen 2021 | 12 | PROSP | MAFLD | 38±9.4 | 92 | 27.1±3.3 | 0 | 17 | 66 | 17 |  | 18F-FDG PET | LIFEx (v6.3) | 60 | LR, SVM, Naïve Bayes, 5-Nearest Neighbor, and discriminant analysis | GLZLM, GLRLM, NGLDM, and GLCM |
| Hu 2022 | 108 | RET | Chronic liver disease |  | 57 |  |  | 25 | 25 | 13 | 22 | CT | syngo.via Frontier Radiomics (v1.2.2) | 1674 | Random forest |  |
| Lan 2019 | 140 | RET | Hepatitis B/C | 51±13 | 56 |  | 20 | 24 | 24 | 11 | 21 | MRE | Analysis Kit | 364 | LR | Histogram parameters, GLCM, and GLRLM |
| Li 2018 | 144 | PROSP | Hepatitis B | 48±14 | 79 | 20.2±3.2 | 10 | 23 | 26 | 16 | 24 | US | Analysis Kit | 396 | AdaBoosting, Decision Tree, Neural Network, Random Forest, LR, and SVM | Conventional radiomics, original radiofrequency data, and Dynamic contrast enhance micro-flow features. |
| Lu 2021 | 807 | RET | Chronic liver disease | 33±10 | 67 | 22±3.3 | 47 | | 22 | 15 | 17 | 2D-SWE |  |  | Deep Learning Radiomics of Elastography |  |
| Park 2019 | 436 | RET | Hepatitis B/C / HCC | 51±15 | 73 |  | 30 | 5 | 12 | 18 | 36 | Gadoxetic acid-enhanced MRI | AsanJ |  | LR | 8 first-order histogram features and higher-order textural features, including 24 GLCM and 11 GLRLM features |
| Qiu 2002 | 74 | RET | Early-stage cirrhosis |  | 0 |  |  |  |  |  |  | DWI | Slicer Radiomics Extension in 3D Slicer (v4.8.1) | 279 | SVM | 18 first-order, intensity histogram-based features and 75 textural features including 24 GLCM, 16 GLRLM, 16 GLSZM, five NGTDM, and 14 GLDM features. |
| Sim 2022 | 100 | RET | MAFLD | 48±13 | 66 | 27±5 |  |  |  |  |  | MRE | PyRadiomics (v3) | 833 | Random Forest | 14 features related to shape, 18 first-order statistical features, and 73 second-order statistical including textural features; GLCM, GLRLM, GLDM, GLSZM, and NGTDM. |
| Wang 2019 | 266 | PROSP | Hepatitis B | 39±12 | 67 | 22.8±3.3 | 16 | | 27 | 32 | 25 | 2D-SWE |  |  | Deep Learning Radiomics of Elastography |  |
| Wang 2022 | 433 | RET | Hepatitis B/C | 59 (49-67) | 80 |  |  |  |  |  |  | CE-CT | Pyradiomics (v2.2.0) | 837 | LASSO regression, SVM |  |
| Xue 2020 | 466 | RET | Chronic hepatitis B | 55±12 | 76 |  | 21 | 12 | 15 | 12 | 40 | Multimodal US |  |  | Inception V3 Network | Gray scale modality and elastography modality images |
| Yin 2022 | 252 | RET | Chronic liver disease | 59 (IQR 48-65) | 56 |  | 53 | 3 | 4 | 7 | 32 | CT | Pyradiomics (v3.0) | 77 | LR, AdaBoosting, Gradient Boosting, XGBoosting | NGTDM, GLSZM, GLRLM, GLCM |
| Zhang 2023 | 209 | RET | Hepatitis B | 38 (IQR 31-46) | 0 |  | 4 | 34 | 35 | 12 | 15 | MRI | Pyradiomics (version 2.1.1) | 837 | LR | 18 first-order statistics, 75 textural (24 GLCMs, 16 GLRLM, 16 GLSZM-based features, 5 NGTDM, and 14 GLDM), and 744 Laplacian of Gaussian filtered, and wavelet transformed features. |
| Zhao 2022 | 139 | RET | Chronic hepatitis |  | 54 |  | 0 | 30 | 24 | 23 | 23 | MRI | ITK-SNAP (v3.30) | 851 | Boruta, LASSO regression | First order, shape, GLCM, GLRLM, NGTDM, gray-level differential matrix, and wavelet transform features. |
| Zheng 2021 | 132 | PROSP | Hepatitis B | 46±13 | 71 |  | 0 | 23 | 21 | 24 | 32 | MRI | Pyradiomics (v2.1.2) | 1379 | LR, Linear Discriminant, k-Nearest-Neighbor, Gaussian Naive Bayes, Decision Tree, and SVM | 14 common shape features, 18 first-order statistical features related to voxel intensities, and 73 textural features. |
| **Abbreviations:** ^18^F-FDG PET, ^18^F-fluorodeoxyglucose positron emission tomography; 2D-SWE, Two-dimensional shear-wave elastography; CE-CT, contrast enhanced computed tomography; DWI, diffusion weighted imaging; GLCM, Grey Level Co-occurrence Matrix; GLDM, gray-level dependence matrix; GLRLM, Grey-Level Run-Length Matrix; GLSZM, Grey Level Size Zone Matrix; GTDM, Grey-tone difference matrix; LASSO, least absolute shrinkage and selection operator; LR, logistic regression; MAFLD, Metabolic dysfunction-associated fatty liver disease; MRE, Magnetic resonance elastography; MRI, Magnetic resonance imaging; NGLDM, Neighboring Grey Level Dependence Matrix; PROSP, prospective; RET, retrospective; SVM, Support Vector Machine; US, ultrasound. | | | | | | | | | | | | | | | | |

| **Table S2: Quality assessment of the included studies (QUADAS-2)** | | | | | | | |
| --- | --- | --- | --- | --- | --- | --- | --- |
| **Study** | **Risk of bias** | | | | **Applicability concerns** | | |
|  | **Patient selection** | **Index test** | **Reference standard** | **Flow and timing** | **Patient selection** | **Index test** | **Reference standard** |
| Chen 2021 | L | L | L | L | L | L | L |
| Hu 2022 | L | L | L | U | L | L | L |
| Lan 2019 | L | L | L | U | L | L | L |
| Li 2018 | L | L | L | U | L | L | L |
| Lu 2021 | L | L | L | L | L | L | L |
| Park 2019 | L | L | L | L | L | L | L |
| Qiu 2002 | L | L | L | U | L | L | L |
| Sim 2022 | L | L | L | L | L | L | L |
| Wang 2019 | L | L | L | L | L | L | L |
| Wang 2022 | L | L | L | L | L | L | L |
| Xue 2020 | L | L | L | L | L | L | L |
| Yin 2022 | L | L | L | U | L | L | L |
| Zhang 2023 | L | L | L | L | L | L | L |
| Zhao 2022 | L | L | L | U | L | L | L |
| Zheng 2021 | L | L | L | L | L | L | L |
| Legends: H, high risk of bias; L, low risk of bias; U, unclear risk of bias | | | | | | | |

|  |
| --- |
| a |
|  |
| b |
| Figure S1: Graphs showing the outcomes of publication bias assessment |

Figure S2a: A forest graph showing the outcomes of the meta-analysis of accuracy of radiomics in differentiating different stages of fibrosis in training cohorts. Abbreviations in study ID: AB, Adaboost; ER, Echo coplanar imaging; GB, Gradient Boosting; GRE, Gradient recalled echo; LR, logistic regression; XGB, XG boosting.

Figure S2b: A forest graph showing the outcomes of the meta-analysis of accuracy of radiomics in differentiating different stages of fibrosis in test/validation cohorts.

Figure S3a: A forest graph showing the outcomes of the meta-analysis of specificity of radiomics in differentiating different stages of fibrosis in training cohorts. Abbreviations in study ID: DLRE, Deep learning radiomics of elastography.

Figure S3b: A forest graph showing the outcomes of meta-analysis of specificity of radiomics in differentiating different stages of fibrosis in test/validation cohorts. Abbreviations in study ID: DLRE, Deep learning radiomics of elastography.

**Appendix S1**

**Literature search strategy**

“Radiomics AND liver fibrosis OR cirrhosis AND diagnostic accuracy”

“Radiomics AND liver fibrosis AND diagnostic accuracy”

“Radiomics AND liver cirrhosis AND diagnostic accuracy”

“Radiomics AND liver fibrosis OR cirrhosis AND diagnostic sensitivity”

“Radiomics AND liver fibrosis OR cirrhosis AND diagnostic specificity”

“Radiomics AND liver fibrosis OR cirrhosis AND predictive value”

“Radiomics AND Machine learning AND liver fibrosis OR cirrhosis AND diagnostic accuracy”

“Radiomics AND Deep Learning AND liver fibrosis OR cirrhosis AND diagnostic accuracy”

“Radiomics AND Adaptive boosting AND liver fibrosis OR cirrhosis AND diagnostic accuracy”

“Radiomics AND Gradient Boosting AND liver fibrosis OR cirrhosis AND diagnostic accuracy”

“Radiomics AND LASSO regression AND liver fibrosis OR cirrhosis AND diagnostic accuracy”

“Radiomics AND Support Vector Machine AND liver fibrosis OR cirrhosis AND diagnostic accuracy”

“Radiomics AND Discriminant analysis AND liver fibrosis OR cirrhosis AND diagnostic accuracy”

“Radiomics AND Decision Tree AND liver fibrosis OR cirrhosis AND diagnostic accuracy”

“Radiomics AND Neural network AND liver fibrosis OR cirrhosis AND diagnostic accuracy”

“Radiomics AND Random Forest AND liver fibrosis OR cirrhosis AND diagnostic accuracy”

“Radiomics AND Grey Level Co-occurrence Matrix AND liver fibrosis OR cirrhosis AND diagnostic accuracy”

“Radiomics AND Grey-level dependence matrix AND liver fibrosis OR cirrhosis AND diagnostic accuracy”

“Radiomics AND Grey-Level Run-Length Matrix AND liver fibrosis OR cirrhosis AND diagnostic accuracy”

“Radiomics AND Grey Level Size Zone Matrix AND liver fibrosis OR cirrhosis AND diagnostic accuracy”

“Radiomics AND Grey-tone difference matrix AND liver fibrosis OR cirrhosis AND diagnostic accuracy”

“Radiomics AND Neighboring Gray Level Dependence Matrix AND liver fibrosis OR cirrhosis AND diagnostic accuracy”
